# Mainstreaming responsible conduct of research education in Malaysia

**DOI:** 10.1080/20961790.2021.1987619

**Published:** 2021-11-05

**Authors:** De-Ming Chau, Lay Ching Chai, Manraj Singh Cheema, Abhi Veerakumarasivam

**Affiliations:** a Department of Biomedical Sciences, Universiti Putra Malaysia, Selangor, Malaysia; b Institute of Biological Sciences, Faculty of Science, University of Malaya, Kuala Lumpur, Malaysia; c School of Medical and Life Sciences, Sunway University, Selangor, Malaysia; d Young Scientists Network-Academy of Sciences Malaysia, Kuala Lumpur, Malaysia

##  

Trust is the bedrock of research, built on honesty, responsibility, accountability, and transparency. When embraced by all researchers and embedded in all aspects of research, these values help ensure that research has a positive impact on the research community, public, and policy makers, thereby enabling discoveries and innovations to enhance the well-being of humanity.

Responsible Conduct of Research (RCR) is a framework that describes ethical values, best practices and professional norms that guide the actions of the research community. It is a vital component of building trust in research. RCR education is essential for training researchers on how to apply this framework and adopt the tools and mindset to make ethical decisions when they face ethical dilemmas in their research [1]. Unfortunately, formal mandatory RCR education is not ubiquitously available in many countries, including Malaysia. As a result, significant gaps in RCR knowledge exist amongst researchers [2]. This ultimately influences the culture of these scientific ecosystems, affecting the ability of these ecosystems to maximise the positive impact of research and development while minimising associated risks.

In recognition of the growing importance of RCR education, the Young Scientists Network-Academy of Sciences Malaysia (YSN-ASM) established the YSN-ASM RCR Programme in 2015 to promote RCR in Malaysia (www.akademisains.gov.my/rcr-book-online). YSN-ASM is the official national young academy that represents top young researchers in Malaysia committed to fostering excellence in science, technology and innovation and advancing this area of the country’s development. This programme traces its genesis to the initial support of the US National Academy of Sciences, Engineering and Medicine (US NASEM), which provided training and seed funding to a group of young researchers in Malaysia to promote RCR [3].

## Promoting grassroots RCR education in Malaysia

Since its inception, the YSN-ASM RCR Programme has been actively conducting RCR workshops and training RCR instructors. The programme collaborates with various government agencies, universities, research institutions, and professional scientific societies across the country.

Although RCR codes and handbooks written by the international scientific community exist, the Malaysian Educational Module on RCR was specially developed to provide contextualized educational content for the Malaysian research community [4]. The module serves as a reference by providing guidelines, professional norms and best practices that were adopted from international RCR handbooks and contextualised for the local research community by using culturally appropriate names, terms, and scenarios. These scenarios are based on prevalent and contemporary issues and not limited to the life sciences [4,5]. This ensures that the material resonates with a wide range of disciplines and researchers in Malaysia, and that it reflects the increasingly interdisciplinary nature of research. The module also educates RCR trainers, increasing capacity to expand awareness and skill in RCR instruction in Malaysia. Instructors who wish to teach RCR can use the step-by-step guidelines, all of which are grounded in active learning pedagogy. In this way, they can develop skills in leading the case studies, facilitating role plays and discussion, and conducting formative and summative assessments. Instructors also have access to participant handouts, model answers and responses to questions posed during discussion sessions.

## Targeting early-career researchers & contextualising active learning pedagogy

These workshops are open to researchers in all stages of their research careers, but emphasis is placed upon early- or mid-career researchers. Although these researchers play a critical role in the advancement of science, technology and innovation in their respective countries, they face significant challenges and unique circumstances that influence their attitudes and practice [6]. Since it is important to imbue RCR culture early, graduate students also attended these workshops. This educational programme consists of 10 curriculum topics: 1) ethical values and responsibilities of researchers, 2) research misconduct, 3) culture of safety and dual use research, 4) conflicts of interest, 5) authorship and publications, 6) peer review, 7) research data management, 8) financial responsibilities, 9) mentor-mentee relationships, and 10) collaborative research. Depending on the number of topics covered, these workshops are typically conducted over a one- to four-day period.

One of the key features of the YSN-ASM RCR Programme is the use of active learning pedagogy. This method of teaching RCR was introduced in Malaysia by the US NASEM RCR trainers at the inaugural institute [3]. Active learning pedagogy is learner-centered and aims to engage the learners’ higher-order cognitive skills. Studies have shown that active learning is an effective teaching pedagogy [7]. In these workshops, participants are encouraged to proactively immerse themselves in the learning of the RCR content through self-reflections and co-learning among participants. The role of the workshop instructor is to facilitate the peer-to-peer learning process through the use of various active learning methods. Since the beginning of the COVID-19 pandemic, we have adapted the RCR workshops to an online format, using tools such as Kahoot®, Jamboard®, and Mentimeter® for brainstorming, and formative assessment to create a dynamic online learning environment.

Whether face-to-face or online, we use case studies with fictional characters that are familiar to the participants. These case studies are written to reflect real-life situations in the Malaysian research and development ecosystem that are complicated by various ethical dilemmas to which researchers can easily relate. While the types of research misconduct and detrimental research practices are universally prevalent, there are nuances that are specific to culture and geography. Cultural appropriateness of case studies is key to ensuring that learning outcomes are achieved [8]. These case studies are accompanied by a series of thought-provoking discussion questions to stimulate internal reflections and sharing among participants. For example, workshop participants are asked to provide an overview of the case, explain the possible motivations behind the action of the characters, describe the nature of the responsible or irresponsible conduct, discuss the consequences of specific actions, and provide strategies to reward positive behaviours and mitigate or prevent irresponsible actions in the future.

Another common active learning method in our workshop is role play. Like the case studies, fictional and relatable scenarios have been created to describe a story with various ethical dilemmas involving multiple actors with competing interests or biases. Role play encourages the participants to empathise with these actors, even if their actions are deemed inappropriate or wrong. By placing themselves in the shoes of these actors, they experience the complex nature of the decision-making process and the gravity of the consequences of their actions. Through these case studies and role playing, participants reflect on similar situations they may have encountered and identify opportunities to improve management of the situation at an individual and institutional level. These activities also offer ideal simulation exercises for early-career participants who may not have previously experienced these situations, helping them prepare to deal with similar scenarios in the future.

## Promoting ethical reflexivity for attitude change

Because of the active-learning pedagogy and contextualized content, workshop participants frequently express that these RCR workshops were very engaging and challenged them to think more profoundly about their social and professional responsibilities as researchers [5]. Often these workshops highlight aspects of RCR that are not vigorously discussed within the research community, providing an opportunity to dispel misconceptions about the various dimensions of research ethics. This is partly due to the fact that the majority of researchers in Malaysia, as in many countries, have not undergone formal RCR training as part of their undergraduate or postgraduate training [2].

More importantly, pre- and post-workshop surveys demonstrate that the workshops empowered participants to promote RCR, sparking a desire for formal training in RCR and a willingness to blow the whistle on RCR transgressions [5].

## Application of RCR to all disciplines

Attention to differences across disciplines is of course critical to providing relevant information and professional standards, but the principles of responsible research span all disciplines. As such, RCR training need not necessarily be discipline specific [9]. The YSN-ASM RCR Programme provides broad educational content, making it suitable and adaptable for teaching RCR to researchers from various fields, including the forensic sciences. Our workshops have engaged those from diverse disciplines, and trainers are encouraged to adapt the materials to their specific field. Case studies and role play scenarios could easily be adapted to those that may arise in forensic sciences research.

Particularly given the highly sensitive nature of many types of forensic sciences research, training in responsible conduct should be an integral component of the curriculum. For any discipline, we recommend an active learning method that facilitates ethical deliberation and self-reflection along the lines of the approach we have used in Malaysia. This approach not only addresses the universal values and norms that are inherent to the responsible conduct of research, but also encourages young researchers to thoughtfully address the many dilemmas and grey zones they will face as they embark upon their research careers.


De-Ming Chau 
*Department of Biomedical Sciences,Universiti Putra Malaysia, Selangor, Malaysia; Young Scientists ­Network-Academy of Sciences Malaysia, Kuala Lumpur, Malaysia*

chau.deming@gmail.com

Lay Ching Chai
*Institute of Biological Sciences, Faculty of Science, University of Malaya, Kuala Lumpur, Malaysia; Young Scientists Network-Academy of Sciences Malaysia, Kuala Lumpur, Malaysia*
Manraj Singh Cheema 
*Department of Biomedical Sciences,Universiti Putra Malaysia, Selangor, Malaysia; Young Scientists Network-Academy of Sciences Malaysia, Kuala Lumpur, Malaysia*
Abhi Veerakumarasivam
*School of Medical and Life Sciences, Sunway University, Selangor, Malaysia; Young Scientists Network-Academy of Sciences Malaysia, Kuala Lumpur, Malaysia*



## References

[1] Responsible conduct of research training. Bethesda (MD): National Institutes of Health, Office of Intramural Research; 2020 Dec 1 [cited 2021 Aug 29]. Available from: https://oir.nih.gov/sourcebook/ethical-conduct/responsible-conduct-research-training

[2] Chau DM, Chai LC, Veerakumarasivam A. Embedding research integrity to ensure quality of high education in Malaysia Proceedings of the Seminar on Internal-External Quality Assurance (SieQA) 2021; 2021 Mar 16–17; Selangor (Malaysia): Sunway Academic Report; 2021; p. 81–91.

[3] An introduction to the U.S. National Academy of Sciences’ educational institutes on responsible science (Kuala Lumpur, Malaysia) [Internet]. Washington, DC: The National Academies of Sciences, Engineering, and Medicine; 2015. [cited 2021 Aug 29]. Available from: http://nas-sites.org/responsiblescience/iircs/institutes/malaysia/

[4] Chau DM, Chai LC, Veerakumarasivam A, editors. Malaysian educational module on responsible conduct of research. Kuala Lumpur (Malaysia): Academy of Sciences Malaysia; 2018.

[5] Chai LC, Chau DM, Veerakumarasivam A. Integrating active learning and content localisation in the delivery of responsible conduct of research education in Malaysia Proceedings of the Seminar on Internal–External Quality Assurance (SieQA) 2021; 2021 Mar 16–17; Selangor (Malaysia): Sunway Academic Report; 2021; p. 13–27.

[6] Geffers J, Beaudry C, Yang HC, et al. Global state of young scientists (GloSYS) in ASEAN–creativity and innovation of young scientists in ASEAN. Halle (Germany): Global Young Academy; 2017.

[7] Freeman S, Eddy SL, McDonough M, et al. Active learning increases student performance in science, engineering, and mathematics. Proc Natl Acad Sci USA. 2014;111:8410–8415. 24821756 10.1073/pnas.1319030111 PMC4060654

[8] Smith-Keiling BL. Intercultural competency: steps for introducing active learning case studies internationally in Confucian heritage culture. J Microbiol Biol Educ. 2019;20:40. 10.1128/jmbe.v20i1.1694 PMC6508903 31160932

[9] Kalichman M, Sweet M, Plemmons D. Standards of scientific conduct: disciplinary differences. Sci Eng Ethics. 2015;21:1085–1093. 25256408 10.1007/s11948-014-9594-0 PMC4375091

